# Conservative Gynecology and Electro-Therapeutics

**Published:** 1899-06

**Authors:** 


**Conservative Gynecology and Electro-Therapeutics.** By G. BETTON MASSEY, M.D. Third Edition. Pp. xv., 394. Philadelphia: The F. A. Davis Company. 1898.

So much new work has been added in this edition of Dr. Massey's book, that it may practically be considered a new work. The author sounds a useful note in stating that every uterine malformation is not a pathological entity requiring treatment; but we must insist that in some pelvic and abdominal cases, and these generally the most grave ones, there will be with the most "conservative" and experienced surgeons an absolute necessity of abdominal section for diagnosis. As a means of diagnosis, the rectal examination of virgins is very properly insisted upon, as it is quite possible to make a complete pelvic examination by this method.

The chapters on the use of electricity in gynæcology are very good, and will repay a careful study; but we cannot agree with the author in all his statements as to the benefits to be derived from this mode of treatment, nor do we consider it safe to employ electricity in certain diseases of the Fallopian tubes or in extra-uterine gestation. The benefit of electricity in fibroid tumours of the uterus is proved most completely, and the various hints given as to the use of the battery, &c., will be of great service to those wishing to practise in this special department.

A striking feature of the volume is the excellence of its illustrations, which include half-tone reproductions of photographs of a professional model, whose services were called into requisition for the delineation of the typical methods of applying electric currents, and also for verifying the motor points of the nerves and muscles.

